# Adenosine A_2A_ Receptors in Bone Marrow-Derived Cells Attenuate Cognitive Impairment in Mice After Chronic Hypoperfusion White Matter Injury

**DOI:** 10.1007/s12975-019-00778-9

**Published:** 2020-05-11

**Authors:** Hong Ran, Jichao Yuan, Jialu Huang, Jie Wang, Kangning Chen, Zhenhua Zhou

**Affiliations:** Department of Neurology, Southwest Hospital, Third Military Medical University (Army Medical University), Chongqing, China

**Keywords:** Adenosine A_2A_ receptors, Bone marrow-derived cells, Cognitive function, Chronic hypoperfusion white matter injury

## Abstract

The mechanism of cognitive dysfunction caused by ischemic white matter lesions is unclear. To explore the effect and mechanism of different cell-derived adenosine A_2A_ receptor (A_2A_R) in cognitive impairment caused by chronic hypoperfusion white matter lesions (CHWMLs), we destroyed the bone marrow hematopoietic capacity of the recipient mice using radiation irradiation followed by establishing the selectively inactivated or reconstituted A_2A_R models with the transplanting bone marrow from global A_2A_R gene knockout or wild-type mice into wild-type or gene knockout mice, respectively. Then Morris Water Maze (MWM), ELISA, immunohistochemistry, and Bielschowsky silver staining were used to assess the effect and mechanism of the cognitive function in chronic cerebral blood flow hypoperfusion (CCH) model. Selectively reconstructing bone marrow-derived cells (BMDCs) A_2A_R (WT → KO group) and activated total adenosine A_2A_R with CGS21680 (CCH + CGS group) improved the cognitive related index. Activation of BMDC A_2A_R suppressed expression of inflammatory cytokines in peripheral blood and reduced the number of activated microglia cells co-localized with cystatin F in local brain, consequently inhibited white matter lesions. On the contrary, selective inactivation of adenosine A_2A_R (KO → WT group) and activation of non-BMDC A_2A_R with CGS21680 (KO → WT + CGS group) served the opposite effects. These results suggested that BMDC A_2A_R could inhibit white matter lesions and attenuate cognitive impairment after CHWMLs, whereas non-BMDC A_2A_Rs aggravate cognitive impairment. The systemic inflammatory response and local activated microglia with cystatin F high expression were involved in the process of cognitive function recovery with BMDC A_2A_R. The overall trend is that BMDC A_2A_Rs play a leading role.

**Electronic supplementary material:**

The online version of this article (10.1007/s12975-019-00778-9) contains supplementary material, which is available to authorized users.

## Introduction

Ischemic leukoencephalopathy refers to a wide range of lesions within the deep perforating arteriole in the hemispheres. The cognitive dysfunction caused by ischemic leukoencephalopathy is the main cause of vascular dementia [1]. Amelioration of cognitive impairment caused by persistent hypoperfusion can significantly improve the survival quality of chronic leukodystrophy patients [2]. However, at present, there is insufficient understanding of the pathogenesis of cognitive dysfunction caused by chronic hypoperfusion white matter lesions (CHWMLs), which greatly hinders the development of therapeutic drugs.

In recent years, the adenosine A_2A_ receptor (A_2A_R) has been extensively studied in various disease models due to its multiple biological effects, such as pro-inflammation, anti-inflammation, neuroprotection [3–5]. In central nervous system diseases, adenosine A_2A_R has a dual role, with a protective role in some diseases [6–8] and aggravation of damage in other diseases [9, 10]. The effect of adenosine A_2A_R on cognitive function is also contradictory. It has been reported that inhibition of neuron adenosine A_2A_R can improve cognitive dysfunction caused by Parkinson’s disease [11]. Conditional knockout of astrocyte A_2A_R can promote long-term memory retention in Alzheimer’s disease patients [7]. Adenosine A_2A_R aggravates intracranial inflammation through activating microglia, leading to cognitive dysfunction in mice with acute ischemia and hypoxia [12]. However, it has also been reported that promoting adenosine A_2A_R accumulation in the hippocampus can promote cognitive improvement in schizophrenia [13], that adenosine A_2A_R knockdown aggravates the motor and cognitive dysfunction of Huntington’s disease by reducing the expression of morphine peptides in the striatum region, and that increasing A_2A_ receptor expression in peripheral blood mononuclear cells can improve the prognosis of mild cognitive function disorder patients by reducing inflammation [14]. These data suggest that adenosine A_2A_R plays two distinct roles in the recovery of cognitive function in different diseases. However, the role and mechanism of adenosine A_2A_R in cognitive dysfunction induced by CHWMLs has not been reported, and it is worthy of further exploration.

Our previous research confirmed that BMDC A_2A_Rs can inhibit inflammation [15], while non-BMDC A_2A_Rs activate microglia and promote inflammatory responses [16]. Different sources of adenosine A_2A_R play contradictory roles. In general, the anti-inflammatory effect mediated by BMDC A_2A_Rs is stronger than the pro-inflammatory effect of non-BMDC A_2A_Rs. Promoting overall adenosine A_2A_ expression can inhibit inflammation and reduce CHWMLs [17]. However, in the persistent chronic hypoperfusion model, the role of different cell-derived adenosine A_2A_R in cognitive function recovery and its mechanism need to be further studied. In consideration of the effect of the inflammation in the cognitive function recovery, we hypothesize that BMDC A_2A_Rs could improve the cognitive function of chronic white matter ischemia model and non-BMDC A_2A_Rs served the opposite effects.

Therefore, the aims of our study were to apply bone marrow transplantation after irradiating wild-type mice and A_2A_ knockout mice to establish models with selective inactivation or reconstruction of BMDC A_2A_Rs, to explore the effect and mechanism of adenosine A_2A_R from different tissue sources on cognitive function in CHWMLs, and to find corresponding targets to improve the quality of life of patients with chronic white matter ischemia.

## Methods

### Experimental Animals and Drugs

Global Adenosine A_2A_R gene knockout (A_2A_R gKO) C57BL/6 mice were a gift from Dr. Jiang-Fan Chen (Boston University School of Medicine, Boston, MA). All mice in this experiment were 8–9 weeks old and weighed 23–30 g. Female mice were donors, and male mice were recipients. The adenosine A_2A_R-specific agonist CGS21680 was purchased from TOCRIS, UK.

### Bone Marrow Cell Extraction

Adenosine A_2A_R KO mice and littermate WT mice were sacrificed by cervical dislocation, and the femur and tibia were separated on a clean platform. The bone marrow cells in the medullary cavity were washed out with RPMI 1640 medium. The cells were collected, filtered through a 200-mesh sieve, and centrifuged at 1500 rpm for 5 min, and the precipitated cells were washed twice with RPMI 1640 medium, centrifuged at 1500 rpm for 5 min, and pulverized in RPMI 1640 medium to form a cell suspension. The cell concentration was adjusted to 2 × 10^8^/mL for use. The cell viability was checked by 0.2% trypan blue staining, and the number of viable cells was greater than 95% for bone marrow transplantation.

### Establishment of a Chimeric Model of Bone Marrow Transplant Cells

The recipient mice (male) were subjected to whole body gamma irradiation with a total dose of 12.5 Gy from a ^60^Co source, which destroyed the bone marrow hematopoietic capacity. The collected bone marrow cell suspension was injected into the recipient mice via the tail vein (the concentration of bone marrow cells was 2 × 10^8^/mL, 0.3 mL/rat), and the bone marrow cell transplantation was completed [18]. Bone marrow cells of female adenosine A_2A_R KO mice were transplanted into male C57BL/6 mice (KO → WT) to establish a selective inactivated model of BMDC A_2A_Rs; bone marrow cells of female WT mice were transplanted into male C57BL/6 mice as controls (WT → WT); bone marrow cells of female WT mice were transplanted into male adenosine A_2A_ receptor KO mice (WT → KO), which was used as a selective reconstruction model of BMDC A_2A_Rs; and bone marrow cells of female adenosine A_2A_ receptor KO mice were transplanted into male adenosine A_2A_ receptor KO mice (KO → KO), which served as controls. The experiment was carried out 8 weeks after transplantation.

### Establishment of a Chronic Cerebral Blood Flow Hypoperfusion Model

A chronic cerebral blood flow hypoperfusion (CCH) model was established with reference to previously described methods [19]. Anesthesia was achieved with isoflurane inhalation, and a supine position was applied. A median longitudinal incision was made after cervical anterior skin depilation and sterilization, the neck muscle was separated, and the bilateral common carotid artery was fully liberated. Two 3-0 nylon threads were used to loosely wrap the near and far end of the right common carotid artery, and two nylon threads were suspended with a hemostat and placed in a specially designed microspring with an inner diameter of 1.8 mm. Then, the spring coil was carefully placed over the common carotid artery; after 30 min, the spring loop was placed over the left common carotid artery with the same method. In the sham-operated control group, the bilateral common carotid arteries were exposed but not clipped. The skin was sutured and disinfected after surgery. The anal temperature of the mice was maintained at 36.5–37.5 °C during the operation. During the whole operation, the Doppler blood flow meter 414-1 probe was fixed at the junction of the scale and the sphenoid wing above the zygomatic arch to measure the cerebral blood flow and evaluate the modeling.

### Morris Water Maze Task

The learning and memory functions of the mice were evaluated using a Morris Water Maze (MWM) task, which was started on the 12th day before modeling and the 21st day after modeling. A black pool with a diameter of 120 cm, a height of 40 cm, and a depth of 25 cm was selected, and warm water with a temperature of 20–23 °C was infused. The pool was divided into 4 quadrants (NW, NE, SE, and SW), and a platform with diameter of 5 cm was placed at the center of the NE quadrant, 1 cm below the horizontal plane. Visual cues of different shapes were given on the NW- and SE-oriented swimming pool walls. The first day was visible platform training (VP), and a marker was placed over the platform to allow the mouse to identify the platform. The mice were placed into the water in a clockwise direction (beginning from the NW quadrant), and the time required for the mouse to find the platform stay on the platform for 10s (escape latency) was recorded. If the mouse did not find the platform within 120s, the experimenter guided it onto the platform, and it stayed on it for 10s. In this situation, the escape latency was recorded as 120s. The second to fifth days were the acquisition phase (AP). At this stage, the mark was removed from the platform, and the above experiment was repeated to record the escape latency and swimming distance. A probe test (PT) was carried out on the sixth day. At this stage, the platform was removed, and the mice were allowed to enter the water from the NW quadrant. The time in the correct quadrant (SE) and the number of passages through the platform location within the 120s were recorded.

### Immunohistochemistry

At the 2nd week and 4th week after CCH, 5 mice were randomly selected from each group, anesthetized with 1% sodium pentobarbital (60 mg/kg), and perfused with 4% paraformaldehyde. Their brain tissues were taken out after dehydration and then frozen (10 μm thick per section). After the sections were antigen-repaired, permeated, and blocked, they were incubated at 4 °C overnight with the ZO-1 antibody. The sections were then incubated with biotin-labeled secondary antibody and horseradish peroxidase label for 2h at room temperature, and finally, coloration was performed with a DAB kit. Then, the expression of ZO-1 was observed under a microscope. Semiquantitative analysis of staining results was performed using Image Pro Plus software. The Nishigaya method was used [20]. The staining intensity of the WT mouse in the sham group (normal) was rated as 4. It was rated as 3 when it was slightly lower than normal, rated as 2 when moderately lower than normal, rated as 1 when significantly lower than normal, rated as 0 when the negative result was positive, rated as 5 when slightly stronger than normal, rated as 6 when it was moderately stronger than normal, and rated as 7 when it was significantly stronger than normal.

### Measurement of Blood-Brain Barrier Permeability

To analyze the alterations in cerebral vascular permeability, Evans blue dye was used as a marker of albumin extravasation. Briefly, mice were injected with 2% EB (3 mL/kg) via the tail vein 4 weeks after CCH. After an hour, animals were then perfused transcardially with phosphate buffer saline (PBS) to purge the intravascular EB dye. Whole brains were isolated, and the dye was extracted with *N*,*N*-dimethylformamide overnight at 50 °C, followed by centrifugation at 5000 rpm for 20 min. The concentration was determined from the OD (610 nm) values according to the standard curve, and then the EB content in the brain tissue was calculated to evaluate blood-brain barrier (BBB) permeability.

### Immunofluorescence Staining

At the 4th week after CCH, tissue sections were fixed in 4% paraformaldehyde for 30 min, followed by permeabilization with a 0.1% Triton X-100 solution for 30 min and antigen blocking with 5% goat serum at room temperature for 30 min. The sections were incubated with cystatin F (1:50, Santa Cruz Biotechnology, Inc., USA) and CD11b (1:100, Chemicon, Colorado, USA) overnight at 4 °C. Then, staining with a fluorescein-labeled secondary antibody and DAPI was performed in the dark. The cells were finally mounted on glass slides and observed under a laser confocal microscope.

### Enzyme-Linked Immune Sorbent Assay

At the 4th week after CCH, 5 mice were randomly selected from each group, and peripheral blood was taken for an enzyme-linked immune sorbent assay (ELISA) to evaluate systemic inflammatory responses. The eBioscience ELISA kit was used, and the reagent instructions were as follows. After preparing the standard, the sample was added and incubated at 37 °C for 30 min. It was then washed repeatedly with PBS buffer, the enzyme standard reagent was added, and the sample then was incubated again at 37 °C for 30 min. Finally, the developer was added, and the absorbance of each well was measured at 450 nm with a microplate reader.

### Bielschowsky Silver Staining

Bielschowsky silver staining was carried out according to the instructions. Paraffin sections were dewaxed, 20% silver nitrate solution was dip-coated for 24 h at room temperature, the sections were washed with double distilled water, 10% formaldehyde was reduced, and then 20% silver nitrate solution + ammonia water was applied again for coloration. The sections were then fixed with 5% sodium thiosulfate and sealed after anhydrous ethanol dehydration. The judgment criteria were graded for the degree of damage of white matter nerve fibers [21]: grade 0, normal; grade 1, disordered arrangement of nerve fibers; grade 2, significant vacuolation (nucleus damage); and grade 3, loss of myelinated nerve fibers (neural fiber axon damage).

### Statistical Methods

All data are presented as the mean ± standard error. Data analysis was performed using SPSS 22.0. A one-way analysis of variance with the appropriate LSD post hoc test was used for comparison of experimental groups. The MWM test results were analyzed with multivariate analysis of variance with the appropriate LSD post hoc test. *P* < 0.05 indicated statistical significance.

## Results

### Adenosine A_2A_R Gene Knockout Had No Effect on Cognitive Function in Normal Mice

**Fig. 1:**
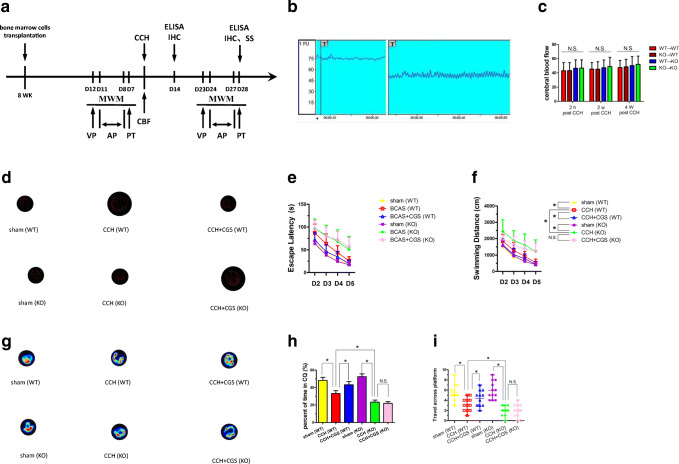
Adenosine A_2A_R knockout aggravates cognitive function impairment in CCH mice. **a** Experimental time axis. **b** Doppler blood flow was used to monitor the changes in cerebral blood flow (CBF) before modeling and 2 h after modeling. CBF reduction rate (%) = (basic CBF value − post ischemic CBF value) / base CBF value × 100. CBF of bone marrow transplantation mice (**c**) was statistically analyzed. **d** The typical trajectory of each group of mice to reach the hidden platform. **e** Escape latency. **f** Swimming distance. **g** The typical heat map of mice in each quadrant. **h** Time in the correct quadrant (SE, lower right quadrant). **i** The number of passages through the platform location. *P < 0.05, N.S., no statistical differences, *N* = 12/group. CBF, cerebral blood flow; CCH, chronic cerebral blood flow hypoperfusion; MWM, Morris water maze task; VP. visible platform training; AP, acquisition phase; PT, probe test

Doppler blood flow monitoring (Fig. 1b) showed the significant differences in the reduction of the cerebral blood flow (CBF) after CCH induction among the groups at each detection time point (Fig. 1c) (*P* > 0.05). Mouse learning and memory functions were assessed with MWM. The AP stage and the PT stage were used to detect the learning ability and memory function, respectively. After systematic training, the time for both sham (WT) and sham (KO) mice to find the platform hidden was gradually shortened (Fig. S1A). There was no statistical difference in the escape latency (Fig. S1A), the swimming distance (Fig. S1B), the time in correct quadrant (CQ) (Fig. S1C), and the number of passing through platform (Fig. S1D). These data illustrated that the A_2A_R knockout had no effect on the motor and cognitive function in normal mice.

### Adenosine A_2A_R Alleviated Cognitive Impairment in CCH Mice

The escape latency in CCH group was significantly prolonged than that in sham group (Fig. 1e), indicating that CHWMLs caused abnormal learning function. The escape latency of the CCH (WT) group was shorter than that in CCH (KO) group, whereas was longer than that in the CCH + CGS (WT) group (Fig. 1e), demonstrating that A_2A_R attenuates cognitive impairment. Although the swimming distance (Fig. 1f) was statistically significant between CCH (WT) and CCH (KO) group, the swimming speed of each group showed no statistical significance, which indicated that A_2A_R inactivation had no effect on motor function. In the space exploration experiment, the time in target quadrant (Fig. 1h) and the number of passing through platforms (Fig. 1i) in CCH (WT) group were statistically significant when compared with these in the CCH + CGS (WT) group and CCH (KO) group. The above data illustrated that CHWMLs led to cognitive dysfunction, and A_2A_R alleviated cognitive impairment induced by CHWMLs.

### Bone Marrow Transplantation Has No Effect on Cognitive Function in Mice

After bone marrow cell transplantation, the time to find the platform hidden underwater in the groups of WT → WT, KO → WT, WT → KO, and KO → KO was gradually shortened, and there was no significant difference in escape latency (Fig. S1E), indicating that the bone marrow transplantation had no effect on the learning ability of normal mice. There was no significant difference in swimming distance (Fig. S1F), in the time in the correct quadrant (Fig. S1G), or the number of passages through the platform location (Fig. S1H) in each group. The above data indicated that bone marrow transplantation had no effect on learning and memory function in mice.

### Selective Inactivation of BMDC A_2A_Rs Aggravates Cognitive Impairment and Reconstructed BMDC A_2A_Rs Promote Cognitive Recovery

**Fig. 2:**
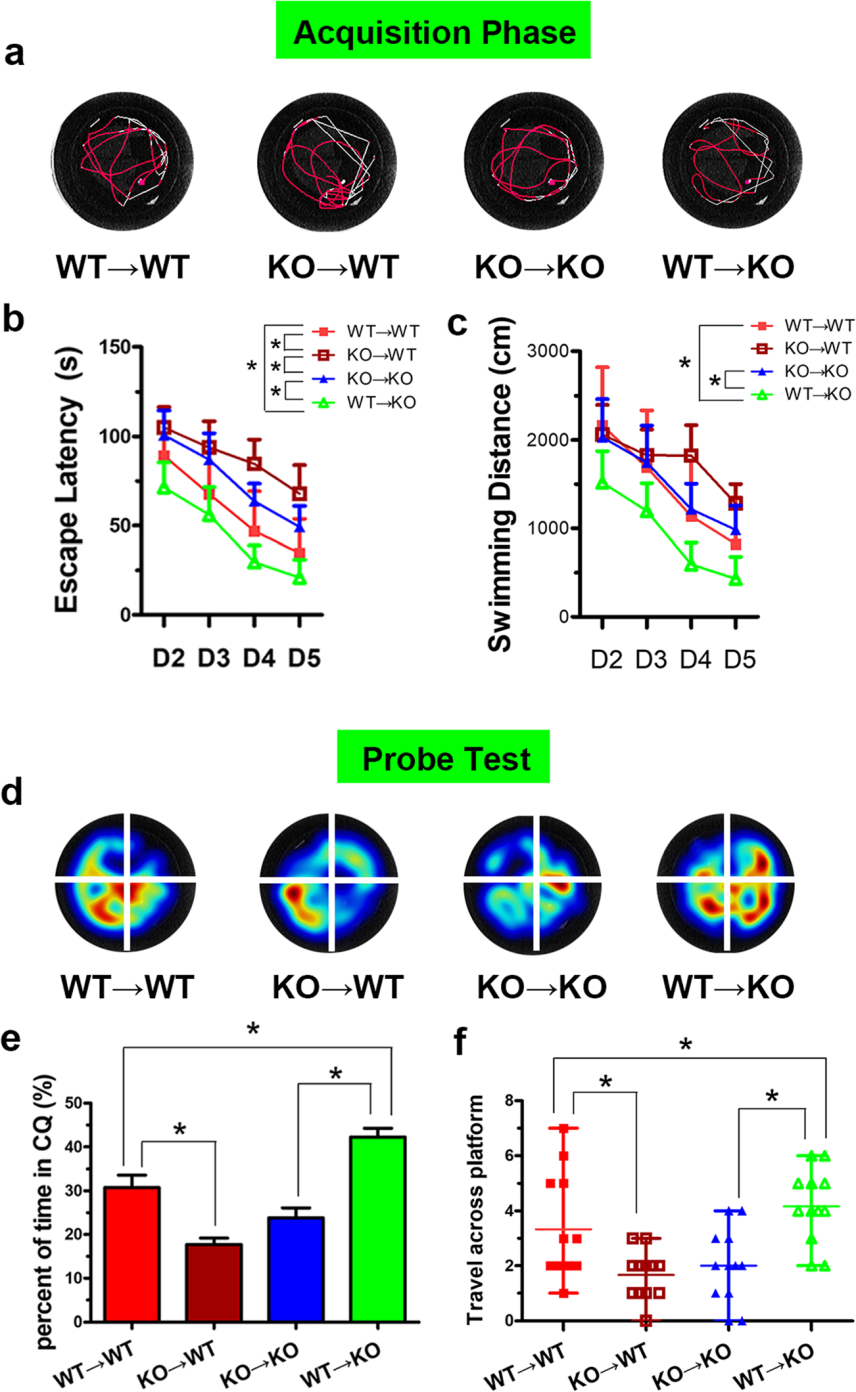
BMDC A_2A_Rs promoted cognition recovery. **a** The typical trajectory of mice to reach the hidden platform. **b** Escape latency. **c** Swimming distance. **d** The typical heat map of mice in each quadrant. **e** Time in the correct quadrant of each group. **f** The number of passages through the platform location. *P < 0.05, *N* = 12/group

To further investigate the role of different cell-derived adenosine A_2A_R in cognitive impairment following CHWMLs, we evaluated the effect of BMDC A_2A_Rs and non-BMDC A_2A_Rs on cognitive recovery by irradiation and bone marrow transplantation. Compared with the WT → WT group, the escape latency of the KO → WT group was longer, the time in the target quadrant was shortened, and the number of passages through the platform location was reduced, indicating that the selective deletion of BMDC A_2A_Rs aggravated cognitive impairment. At the same time, the cognitive related indexes in the WT → KO group were also significantly different from those in the KO → KO group (Fig. 2). These data indicated that BMDC A_2A_R reconstruction can promote cognitive function recovery after CHWMLs.

### Non-BMDC A_2A_R Activation Aggravates Cognitive Impairment

**Fig. 3:**
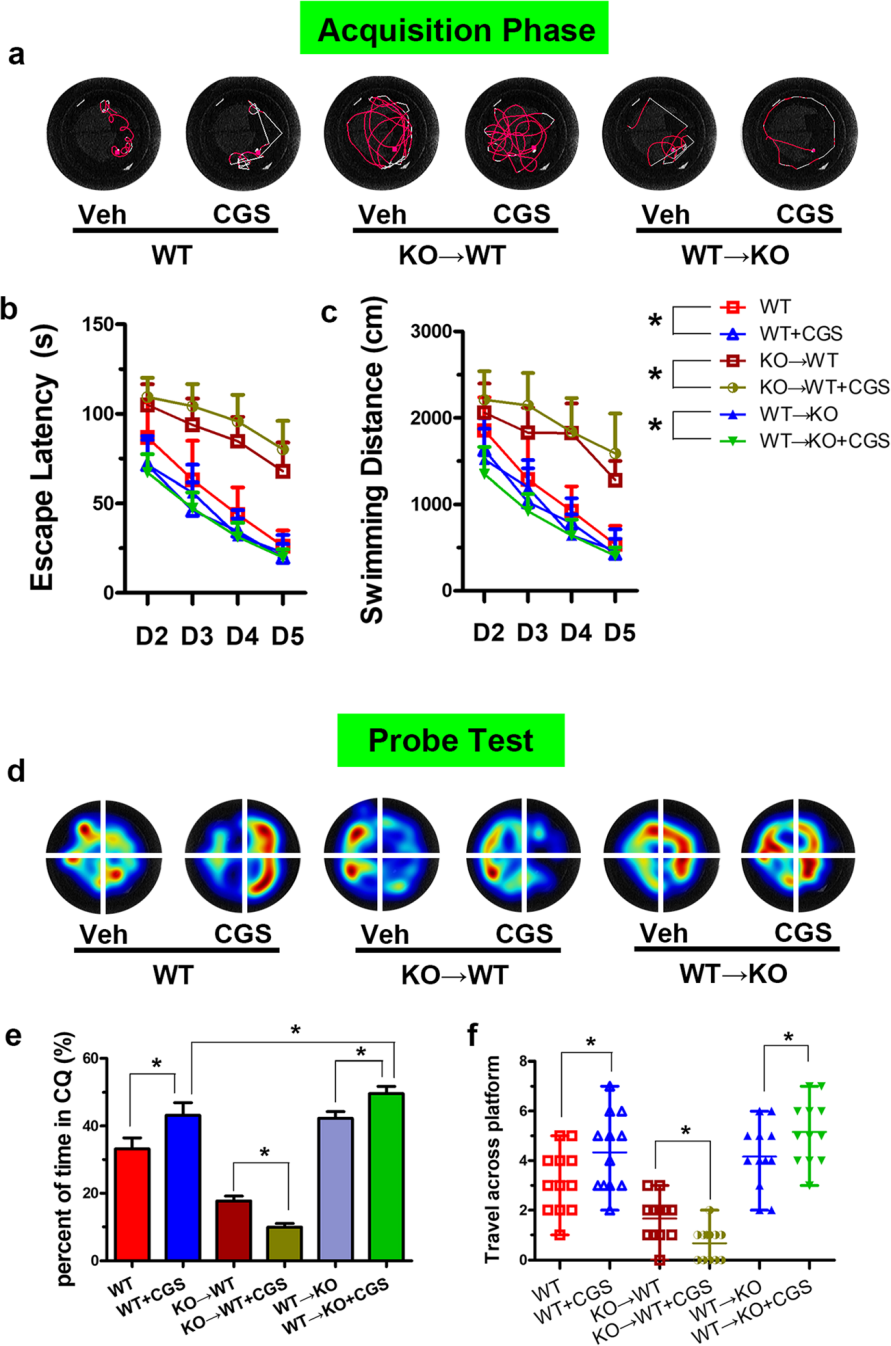
Non-BMDC A_2A_R activation aggravates cognitive impairment. **a** The typical trajectory of each group to reach the hidden platform. **b** Escape latency. **c** Swimming distance. **d** The typical heat map of mice in each quadrant. **e** Time in the correct quadrant of each group. **f** The number of passages through the platform location. *P < 0.05, *N* = 12/group

Based on the analysis of the experimental results, we found that the cognitive function-related indexes in the KO → WT group were significantly different from those in the WT → KO group (Fig. 2). These data suggested that non-BMDC A_2A_Rs may aggravate cognitive impairment. To further validate this conclusion, we injected CCH mice with the adenosine A_2A_R agonist CGS21680. Compared with the KO → WT group, the KO → WT + CGS group had a prolonged escape latency, shortened time in the target quadrant, and reduced number of passages through the platform location (Fig. 3). These data fully demonstrated that non-BMDC A_2A_R activation aggravates cognitive impairment caused by CCH.

### BMDC A_2A_Rs Play a Leading Role in the Recovery of Cognitive Function After CHWMLs

Different sources of adenosine A_2A_R play different roles in cognitive function recovery. Compared with the WT → KO group, the escape latency was shortened, the time in the target quadrant and the number of passages through the platform location increased in the WT → KO + CGS group (Fig. 3), suggesting that activation of BMDC A_2A_Rs promoted cognitive recovery. The MWM task results in the KO → WT group were significantly different from those in the KO → WT + CGS group, suggesting that further activation of non-BMDC A_2A_Rs aggravated cognitive impairment. The escape latency, time in the target quadrant, and number of passages through the platform location in the WT + Veh group were significantly different from those in the WT + CGS group, indicating that BMDC A_2A_Rs play a leading role in cognitive function recovery.

### BMDC A_2A_Rs Suppressed Systemic Inflammation and Non-BMDC A_2A_Rs Aggravated Systemic Inflammatory Response in CHWMLs

**Fig. 4:**
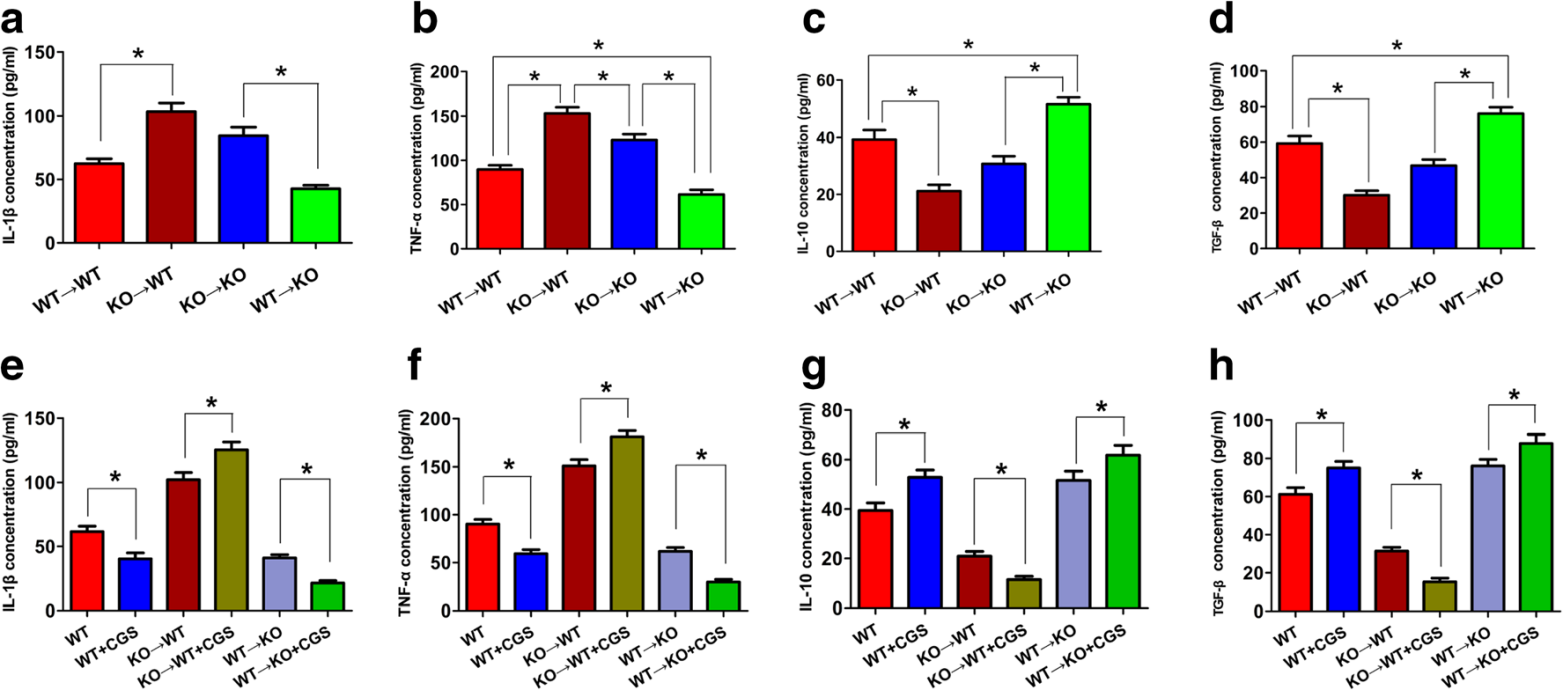
BMDC A_2A_Rs suppress systemic inflammatory responses. The expressions of IL-1β (**a**, **e**), TNF-α (**b**, **f**), IL-10 (**c**, **g**), and TGFβ (**d**, **h**) were detected with an ELISA. *P < 0.05, *N* = 5/group

We subsequently evaluated the mechanism by which BMDC A_2A_Rs promoted cognitive function recovery after CHWMLs. Systemic inflammatory responses in each group were measured with an ELISA (Fig. 4). Compared with the WT → WT group, the expression of the pro-inflammatory cytokines IL-1β and TNF-α was increased, and that of the anti-inflammatory cytokine IL-10 and TGFβ was decreased in the KO → WT group, indicating that the selective inactivation of BMDC A_2A_Rs leads to a systemic inflammatory reaction. The expression of inflammatory factors in WT → KO and KO → KO groups was significantly different, indicating that selective reconstitution of BMDC A_2A_Rs can inhibit inflammation. The expression of pro-inflammatory cytokines IL-1β and TNF-α in the KO → WT group was higher and the expression of anti-inflammatory cytokines IL-10 and TGFβ was less than that in the KO → KO group, indicating that non-BMDC A_2A_Rs promoted an inflammatory response.

To confirm the effect of adenosine A_2A_R from different tissue sources on the inflammatory response, we used the A_2A_R-specific agonist CGS21680 to intervene in different bone marrow cells of transplanted mice. Compared with the WT → KO group, the anti-inflammatory cytokines were suppressed and the pro-inflammatory cytokines were increased in the WT → KO + CGS group, indicating that activation of BMDC A_2A_Rs can inhibit the inflammatory response. The expression of inflammatory factors in the KO → WT group was also significantly different from that in the KO → WT + CGS group, further suggesting that non-BMDC A_2A_Rs aggravated the inflammation. IL-1β and TNF-α expressions in the WT + CGS group were higher, and IL-10 and TGFβ were decreased compared with the WT + Veh group, indicating that the anti-inflammatory effect of BMDC A_2A_Rs plays a leading role in CHWMLs.

### BMDC A_2A_Rs Inhibited Microglia Activated in CHWMLs

Firstly, we examined the effect of adenosine A_2A_R on BBB integrity. We found that A_2A_R knockout (Fig. S2A, B) and bone marrow transplantation (Fig. S2C, D) had no effect on the BBB dysfunction in normal mice. The ZO-1 immunohistochemistry staining and Evans blue content test demonstrated that the BBB was destroyed and gradually repaired after CCH. However, there was no statistical difference in ZO-1 expression and Evans blue content among each group with different interventions (Fig. S2) at each time point. These data indicated that BBB alterations were not the key points for cognitive function recovery with adenosine A_2A_R.

**Fig. 5:**
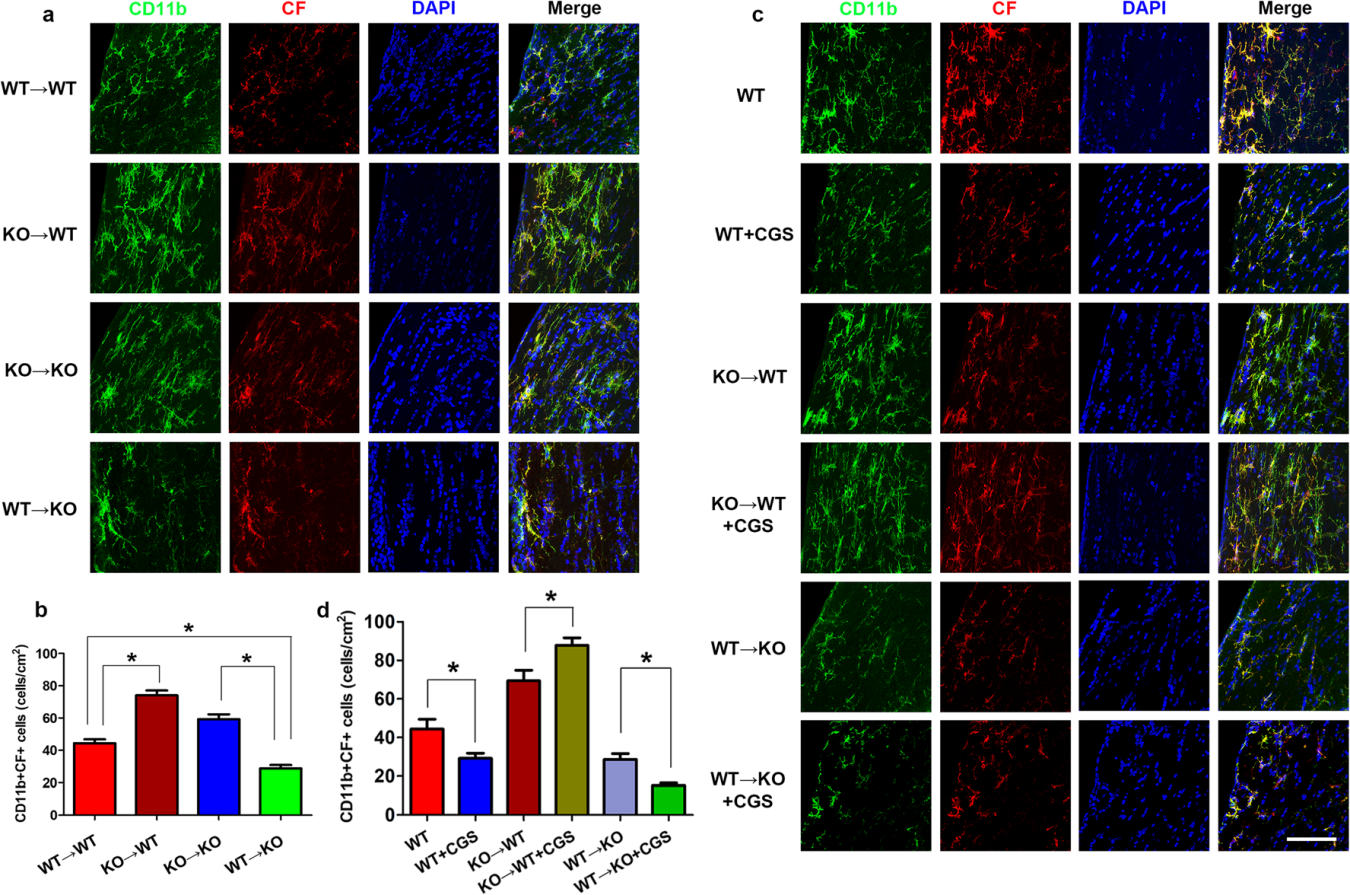
BMDC A_2A_Rs inhibited CF-mediated microglia activation 4 weeks post CCH. **a** Confocal microscopy demonstrating the expression of CF co-localization with microglia after selectively inactivated or reconstituted A_2A_R in BMDCs. The activated microglia marker CD11b was labeled with Alexa Fluor 488 (green), the CF was stained with Alexa Fluor 555 (red), and nuclei were stained with DAPI (blue). **b** Quantitative analysis for the number of CF+CD+ cells after selectively inactivated or reconstituted A_2A_R in BMDCs. Confocal microscopy (**c**) and quantitative analysis (**d**) for CF+CD+ cells with adenosine A_2A_R agonist CGS21680 intervention. *P < 0.05, bar = 20 μm, *N* = 5/group

Adenosine A_2A_R involves in immune response during inflammation and infection. In addition, we also reported that cystatin F (CF) in the activated microglia was closely associated with the effect of the A_2A_R [17]. Therefore, we subsequently investigated whether CF was implicated in the cognitive function recovery mediated with adenosine A_2A_R (Fig. 5). Immunofluorescence staining revealed the expression of CF was co-localized with CD11b, a specific biomarker of microglia after CCH in WT → WT group. A large number of CF+CD11b+ cells were observed in BMDC A_2A_Rs selective inactivated group (KO → WT). By contrast, BMDC A_2A_Rs reconstruction group (WT → KO) significantly reduced the number of CF+CD11b+ cells, compared to the KO → KO group. Activated BMDC A_2A_Rs with CGS administration could decrease the number of CF+CD11b+ cells, whereas activated non-BMDC A_2A_Rs with CGS increase CF+CD11b+ cells. These data suggested that CF-mediated neuroinflammation involved in cognitive function recovery with adenosine A_2A_R.

To further evaluate the mechanism by which BMDC A_2A_Rs promote cognitive function recovery after CHWMLs, we examined the effect of A_2A_R on blood-brain barrier repair. There was no significant difference in ZO-1 expression between the WT group and KO group (Fig. 4a, b) and no significant difference among the WT → WT, KO → WT, WT → KO, and KO → KO groups (Fig. 4c, d), indicating that adenosine A2AR gene knockout and bone marrow transplantation had no effect on the blood-brain barrier of normal mice. Two weeks after CCH, the blood-brain barrier was destroyed, but there was no significant difference in ZO-1 expression among the groups (Fig. 4e, f). The expression of ZO-1 increased at 4 weeks post CCH compared with that at 2 weeks after modeling, indicating that the blood-brain barrier continued to recover, whereas there was no significant difference in ZO-1 expression among the groups (Fig. 4g, h). These data indicated that adenosine A_2A_R had no effect on recovery of the blood-brain barrier.

### Selective Inactivated BMDC A_2A_Rs Aggravated White Matter Lesions

**Fig. 6:**
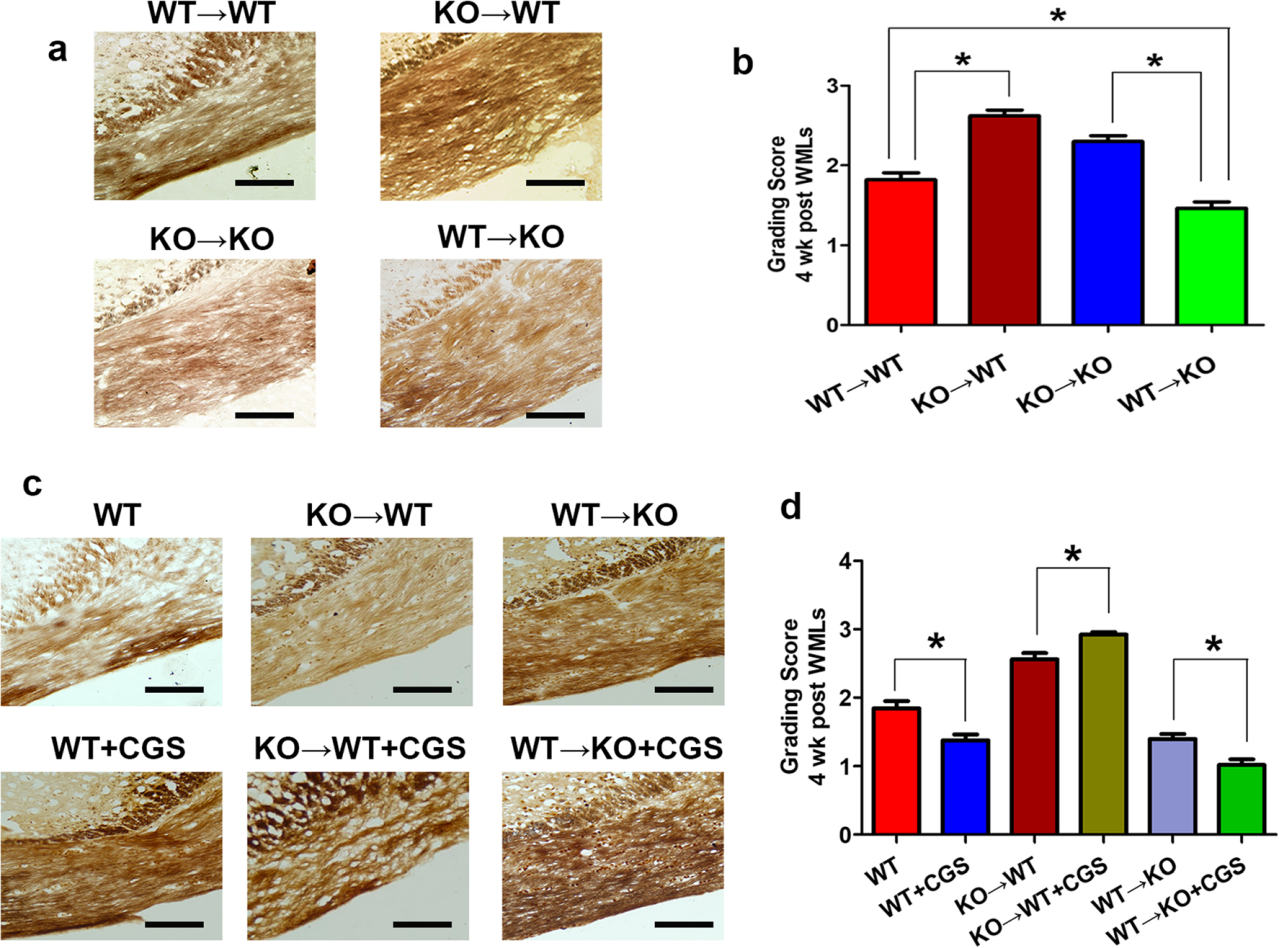
BMDC A_2A_R reduced white matter lesions. **a** Bielschowsky silver staining results of BMDC A_2A_R selective inactivated or reconstruction group. **b** Statistical analysis of silver staining in each group. **c** Bielschowsky silver staining results for each group after adenosine A_2A_ receptor-specific agonist CGS21680 intervention. **d** Silver staining statistical analysis with CGS21680 intervention. *P < 0.05, bar = 20 μm, *N* = 5/group

Bielschowsky silver staining was used to assess white matter lesions in the corpus callosum (Fig. 6). Under normal circumstances, the nerve fibers around the corpus callosum are arranged closely and orderly. After chronic ischemia, the nerve fibers are disordered, suggesting there was white matter damage. The statistical results in the bone marrow cell transplant groups indicated that the degree of corpus callosum white matter nerve fiber damage was ordered from high to low as follows: KO → WT, KO → KO, WT → WT, WT → KO. The silver staining score of the A_2A_R-specific agonist CGS21680 intervention group had a statistically difference compared with other groups. These results suggested that BMDC A_2A_RsR knockout aggravated demyelination, the non-BMDC A_2A_R knockout exerts the opposite effect, and the role of BMDC A_2A_Rs is dominant.

## Discussion

Our results confirmed that BMDC A_2A_Rs promoted cognitive function recovery in a CCH mouse model, while non-BMDC A_2A_Rs aggravated cognitive impairment. In general, the function of BMDC A_2A_Rs to promote cognitive recovery plays a leading role, and its mechanism is mainly achieved by reducing inflammation and white matter lesions.

Dementia caused by vascular cognitive impairment accounts for more than 20% of the number of dementia patients, which is the second leading cause of dementia, after Alzheimer’s disease (AD) [22, 23]. Chronic hypoperfusion is an important factor leading to vascular cognitive impairment, and it has received increasing attention in recent years. The phosphodiesterase inhibitor roflumilast [24] and the transient receptor potential melastatin 2 (TRPM2) inhibitor [25] can suppress microglial activity after CCH injury and promote cognitive function recovery through anti-inflammatory effects. IL-1β receptor inhibitors and IL-1β knockout mice block the IL-1β signaling pathway, thereby inducing oligodendrocyte premature cell migration to the periphery of the corpus callosum, and avoiding white matter damage and reversing chronic hypoperfusion-induced cognitive dysfunction by improving the local inflammatory environment [26]. MiR-181c [27] and MiR-96 [28] can ameliorate cognitive dysfunction by regulating autophagy in CCH [29]. In general, demyelination, inflammatory responses, and mitochondrial dysfunction are common pathways leading to cognitive dysfunction [30, 31], which can ameliorate the prognosis of chronic cortical hypoperfusion by reducing white matter lesions.

Adenosine is an intermediate product of energy metabolism, which is widely distributed in various tissues and organs. It is also an important neuromodulator in the nervous system that plays an important role in regulating inflammation. Our previous studies reported that the anti-inflammatory effects of BMDC A_2A_Rs are stronger than the pro-inflammatory effects of non-BMDC A_2A_Rs [15], but it is not clear whether its role in cognitive impairment is caused by CHWMLs. MWM showed that the cognitive function-related indexes in the CCH (WT) group were significantly different from those in the CCH (KO) group. These data suggested that adenosine A_2A_R involved in cognitive impairment after CHWMLs. To further evaluate the role of adenosine A_2A_R in cognitive impairment in different tissue sources, we used irradiation and bone marrow transplantation to establish a selective inactivated or reconstruction model of BMDC A_2A_Rs. The results of the MWM test suggested that after selective deletion of BMDC A_2A_Rs, the escape latency was prolonged, the time in the target quadrant was shortened, the number of passages through the platform location was reduced, and the trend was consistent with the trend of A_2A_R whole gene knockout. The selective reconstruction of BMDC A_2A_Rs can reverse the above results, further indicating that BMDC A_2A_Rs have protective effects on cognitive function. In the course of the experiment, we also found an interesting phenomenon; that is, the cognitive function-related index in the KO → WT group had a significant difference in comparison with the WT → KO group, which suggested that non-BMDC A_2A_Rs may aggravate cognitive function damage. To further confirm this hypothesis, we used the adenosine A_2A_R agonist CGS21680 to interfere in different bone marrow transplantation groups. The experimental results suggested that only activated non-BMDC A_2A_Rs prolong the escape latency, reduce the time in the target quadrant, and decrease the number of passages through the platforms. Only activated BMDC A_2A_Rs will shorten the escape latency and increase the time in the target quadrant and the number of passages through the platform location. These data confirmed our hypothesis that BMDC A_2A_Rs promote cognitive function recovery and that non-BMDC A_2A_Rs aggravate cognitive impairment. However, the overall trend is that adenosine A_2A_R provides a protective effect on cognitive impairment in CHWMLs.

As we previously mentioned, the inflammatory response is the basic pathway for cognitive impairment caused by CHWMLs. We therefore further verified whether anti-inflammation is the mechanism by which adenosine A_2A_R promotes the recovery of neurological function. Selective inactivated BMDC A_2A_Rs or activated non-BMDC A_2A_Rs led to systemic inflammatory response, and increased the expression of CF, accordingly activated microglia, whereas selective reconstitution reversed the above phenomenon. CF, a potent endogenous cysteine protease inhibitor, was substantially up-regulated in regions of white matter rarefaction that occurred in various demyelinating diseases of the CNS [32, 33]. In this study, we found that BMDC A_2A_Rs suppressed CF-mediated microglia activated and non-BMDC A_2A_Rs promoted CF-mediated microglia activated, which was consistent with our previous research [16]. The immunohistochemistry suggested BBB alterations was not the key point for cognitive function recovery with adenosine A_2A_Rs. However, elevated BBB permeability was the pathological basis of systemic inflammatory cytokines acting on brain parenchyma cells. Silver staining results suggested that activation of BMDC A_2A_Rs can alleviate corpus callosum white matter damage. These data confirmed our hypothesis that adenosine A_2A_R promotes the recovery of cognitive function in mice by inhibiting inflammatory responses and reducing white matter lesions. In this process, BMDC A_2A_Rs play a leading role.

In the AD and PD disease models, adenosine A_2A_R aggravates cognitive dysfunction, whereas adenosine A_2A_R can improve cognitive function in the HD and schizophrenia disease models. It is worth further consideration to determine the reason for the exact opposite effect of A_2A_R. Literature reports have suggested that adenosine A_2A_R had two main pathways for regulating cognitive function: one is via integrating dopamine, glutamate, N-methyl-D-aspartate (NMDA), and brain-derived neurotrophic factor signaling pathways, and the other is regulating inflammatory responses [34]. Adenosine A_2A_R mediates cognitive dysfunction in AD and PD disease models mainly through the first pathway [9, 35, 36], while in the HD and schizophrenia disease models, it mainly protects through the second pathway [13, 14, 37, 38]. This study showed that adenosine A_2A_R also improved cognitive function through anti-inflammatory effects in CHWMLs, and this mechanism of action is consistent with the above conjecture. However, at the same time, we must also realize that in other disease models [12, 39–42], the role of adenosine A_2A_R in cognitive function cannot be explained by the above mechanism, indicating that there may be other functional ways, which requires further study.

Although the FDA has approved acetylcholinesterase inhibitors and an NMDA receptor antagonist, memantine, as a drug for the treatment of cognitive dysfunction since 1993, these drugs currently only play a role in delaying the course of cognitive dysfunction and cannot effectively stop or reverse the course of the disease [43]. Moreover, specific drugs for cognitive impairment of chronic hypoperfusion leukoencephalopathy are lacking. Adenosine A_2A_R has multiple biological effects and can play different roles in different disease models. This study found that BMDC A_2A_Rs inhibit cognitive function after CHWMLs through anti-inflammatory effects. Non-BMDC A_2A_Rs can aggravate cognitive impairment through inflammation. Overall, BMDC A_2A_Rs play a leading role. This study initially explored the role and mechanism of adenosine A2AR in the pathogenesis of cognitive impairment in CHWMLs and provides a new treatment strategy for CHWMLs.

## Conclusions

This study finds that BMDC A_2A_Rs inhibit cognitive impairment after CHWMLs through anti-inflammatory effects. Non-BMDC A_2A_Rs can aggravate cognitive impairment through pro-inflammation. Overall, global A_2A_Rs activation could alleviate cognitive impairment after CHWMLs. This study initially explores the role and mechanism of adenosine A_2A_R in the pathogenesis of cognitive impairment in CHWMLs, and provides a new treatment strategy for CHWMLs.

## Electronic Supplementary Material

ESM 1 (PNG 428 kb)

ESM 2 (PNG 290 kb)
