# Supplementary figures and images for: Adenosine A2A Receptors in Bone Marrow-Derived Cells Attenuate Cognitive Impairment in Mice After Chronic Hypoperfusion White Matter Injury
Source: Transl Stroke Res. 2020 May 11;11(5):1028–40. doi: 10.1007/s12975-019-00778-9 (PMC7496018; doi:10.1007/s12975-019-00778-9)

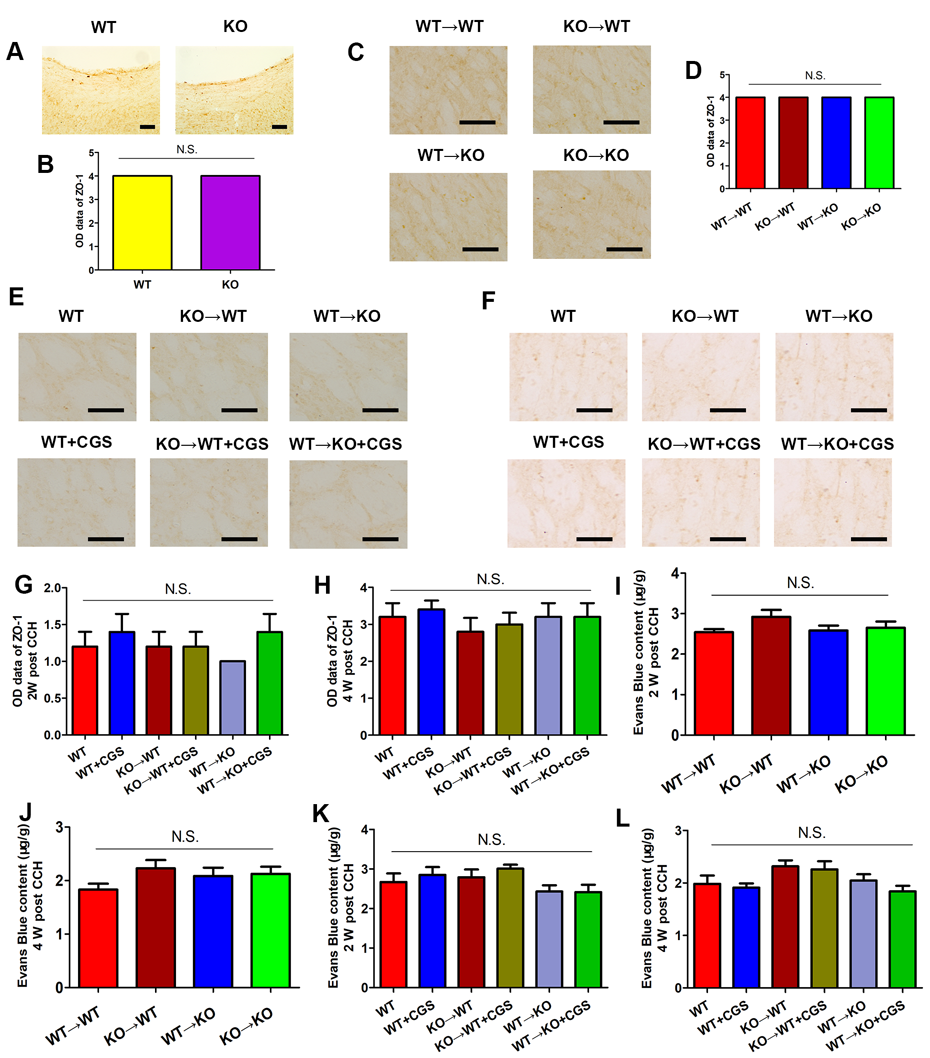

Supplement: Supplementary file 1 — (PNG 428 kb) [file 12975_2019_778_MOESM1_ESM.png]

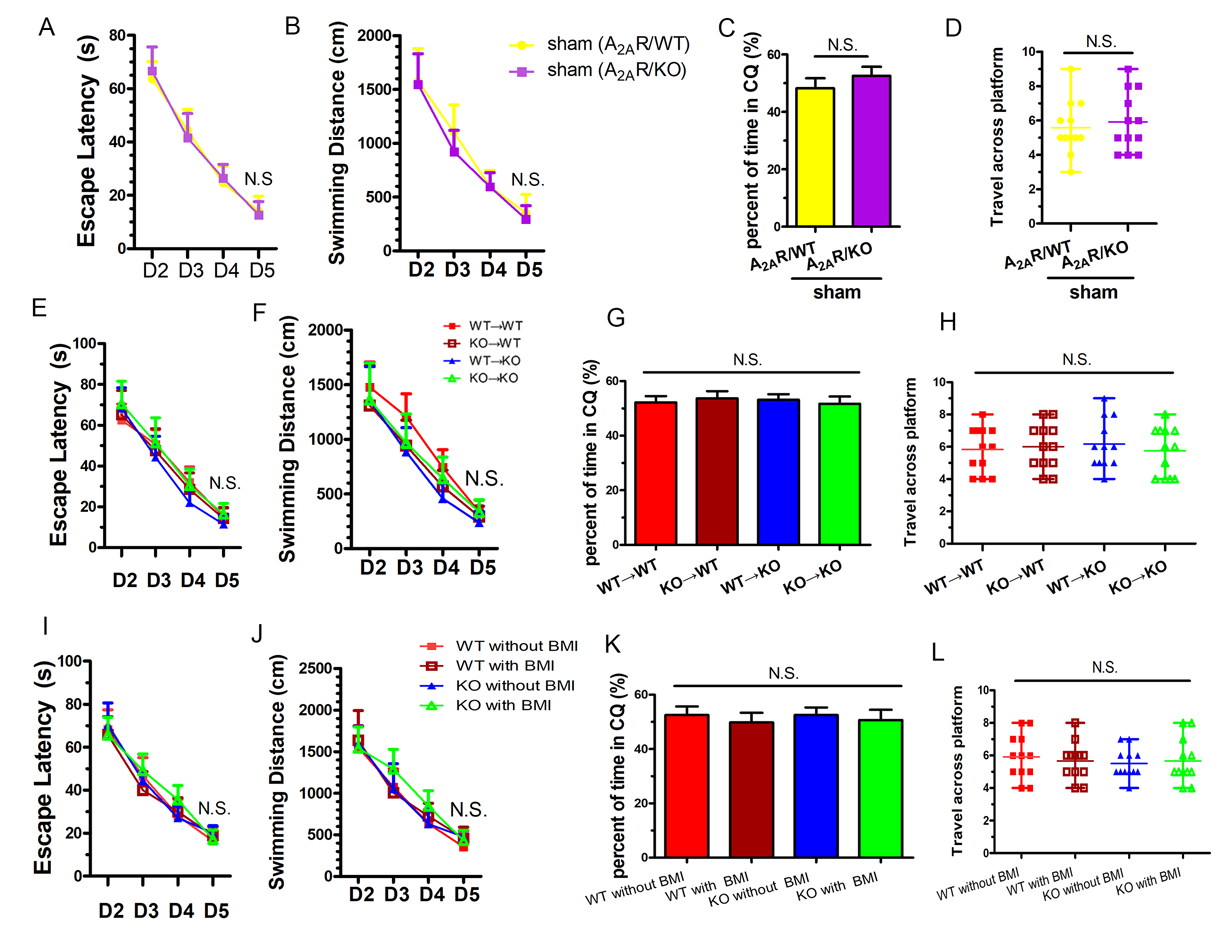

Supplement: Supplementary file 2 — (PNG 290 kb) [file 12975_2019_778_MOESM2_ESM.png]
